# Supplementary material for: Are carbon emissions trading and green financial instruments synergistic? -Comprehensive quantitative research based on content analysis
Source: PLoS One. 2024 Mar 7;19(3):e0298601. doi: 10.1371/journal.pone.0298601 (PMC10919601; doi:10.1371/journal.pone.0298601)
Supplement: S1 Data — (ZIP) [file pone.0298601.s001.zip › Data/地级市Z_F工作报告.zip!/║╙▒▒/║Γ╦«╩╨/└┤╫╘╣1⁄2╓┌║┼í╛┬φ┐╦╩2╛▌═°í┐_2013.docx]

各位代表：

现在，我代表县人民政府向大会作工作报告，请予审议。并请县政协各位委员和列席会议的同志提出意见。

一、过去一年工作回顾

2012年是我们积极应对复杂局面、战胜各种困难挑战、取得重要发展成就的一年。在市委、市政府和县委的正确领导下，在县人大、县政协的监督支持下，以迎接十八大、学习十八大、贯彻十八大为主线，团结和带领全县人民，深入落实科学发展观，着力改善发展环境和生态环境，心无旁骛谋发展，千方百计求突破，较好地完成了县十五届人大一次会议确定的各项目标任务，全县保持了经济快速发展、社会和谐稳定、人民安居乐业的良好局面。

综合实力明显提升。全县生产总值完成77.6亿元，同比增长10.3%；全社会固定资产投资56.8亿元，增长28%；规模以上工业增加值24.3亿元，增长17%；城镇居民人均可支配收入1.6万元，增长13.1%，农民人均纯收入5939元，增长15.4%;外贸出口5.5亿美元，增长38.3%；财政收入5.87亿元，增长32.6%，其中一般预算收入2.59亿元，增长32.8%。枣强经济连续第四年快速增长，县域综合实力迈上新的台阶。

项目建设扎实推进。紧紧抓住百家央企、百家院校进河北、衡水湖国际马拉松赛、大营国际皮草交易会等有利时机，大力开展招商引资活动，新建续建亿元以上项目43个，总投资183.2亿元，列入省、市重点项目8个。大营国际裘都世贸城、瑞星调压器基地等一批重点项目竣工投产。大营国际裘皮新城、恒润集团非稳态管道、欧意电器北方基地等一批重大项目顺利推进。大营国际裘皮新城完成商业面积20万平方米，恒润非稳态管道被列为国家战略新兴产业支持项目。河北纳森空调等一批大项目完成前期准备。全县项目建设呈现出梯次推进、滚动发展的良好局面。

产业升级步伐加快。坚持提高研发能力、推进技术改造、加强品牌建设、培育骨干企业，促进主导产业优化升级。县政府与恒润集团、武汉理工大学合作，启动建设了河北省复合材料产业技术研究院，被列为省重点支持的十家研究院之一；鑫山输送机械公司与北京起重机械设计研究院合作，创建了输送机械制造基地；全国首家国家级裘皮产品检测重点实验室落户大营。全年共实施技改项目25个，申报专利38项，研发新产品12项，6项产品获省著名商标、省名优产品，全县规模以上企业达到109家。皮毛、玻璃钢、机械制造三大产业分别纳税1.76亿元、7891万元、5114万元，同比增长39.7%、39.9%和23%。

园区平台作用凸显。县城工业园区启动建设了一号路，拓宽改造了振华街，打通了建华大街，绿化亮化了东外环、富强北路，园区框架进一步拉大，入园企业达到94家，初步形成了多基复合材料、中央空调成套制品、燃气系列产品、新能源等四大板块。大营工业园区开工建设了北环路、西环路、纬一路、经贸路北段等主干道路，基础设施进一步完善，入园企业达到206家。

城乡建设统筹开展。以创建省级园林城、文明城、卫生城为契机，扎实推进47项重点城建工程。县城西出口、东出口、北出口改造全面实施，和平路、裕华路、建设南路等主干道路改造全面竣工，大广高速连接线绿化、亮化圆满完成。实施了郑昔线、邢德线大修中修工程。新建和翻修了192.3公里乡村道路，硬化了68个村主干街道。集中开展市容卫生、夜景照明、交通秩序等专项整治，县城主次街道亮灯率达到98%以上。大力加强生态建设，新增绿地面积57万平方米，人均公共绿地面积达到10.6平方米，城区绿地率、绿化覆盖率分别达到35.9%和41.5%。大力推进居住小区建设，新增居住面积53万平方米。县城建成区面积达到13.7平方公里，荣获“河北省人居环境进步奖”，成为全市唯一获此奖项的县市。

农村经济稳步发展。认真落实扶农惠农政策，共发放粮食直补、综合直补等各类补贴 1.07亿元。以产业扶贫攻坚为主线，编制完成了《枣强县区域发展和扶贫攻坚规划》，35个村被列入省“十二五”扶贫计划。大力发展高效农业、设施农业、示范农业，粮食生产实现“九连增”，总产量达到35.2万吨，棉花总产量2.6万吨，新增优质林果8360亩，达到9万亩，新发展设施蔬菜2400亩，总面积达到6000多亩。小型农田水利重点县项目扎实推进，共安装防渗管道36万米，建咸淡混浇井组60个。特色养殖业快速发展，新增规模养殖场9个，总数达到65个，毛皮动物存栏28.4万只，出栏62.4万只。大力开展植树造林，全年植树133万株、1.8万亩。农业合作组织健康发展，新增农业专业合作社109家，达到628家；农产品品牌达到69个，“清凉江”牌甘薯等5项农产品获得中国绿色食品、无公害农产品和有机农产品证书，农业产业化率达到63.2%。

社会民生持续改善。把改善民生作为最大责任，努力提高人民群众幸福指数。新增就业岗位1800个，城镇登记失业率3.5%。顺利通过省政府教育督导评估，被评为河北省教育工作先进县。枣中本一上线突破千人大关，升学率再创新高。深入推进“文化资源共享工程”，农家书屋建设实现全覆盖。以范党育同志为原型的《营盘镇警事》在央视播出，精神文明建设取得新成效。县医院、第三中学、特教学校新建工程顺利推进。联村供水工程稳步实施，20万人安全饮水问题得到解决。新建廉租房、经济适用房等各类保障性住房874套，改造农村危房956套，困难群众住房条件明显改善。加强基层建设年活动成效显著，落实帮扶资金5093万元，实施帮扶项目746个，57个村面貌焕然一新。新农合、新农保、新城保参合率、参保率均超过95%，城乡低保对象达到1.01万人。以“党育为民工作站”为平台，大力实施和谐平安工程，探索创新社会管理的新机制，群众工作不断加强。“打非治违”专项行动取得明显成效，全年未发生重特大安全事故。认真抓好衡水湖马拉松赛事、十八大期间安保工作，保持了社会和谐稳定的良好局面。企业改制、节能减排、食品安全等工作顺利推进，人民武装、广播电视、物价、国土、审计、统计、计生、档案等各项社会事业也取得了新成绩。

自身建设全面加强。自觉接受人大法律监督、工作监督、政协民主监督和社会舆论监督，人大代表建议和政协委员提案办复率100%。深入开展以“创先争优”为主题的机关效能建设活动，积极推行电子政务，努力提高政府工作效率。加强廉政建设，强化行政监察和审计监督，加大对不正之风的治理力度，政风建设取得新成效。

各位代表，过去一年，面对的形势比预计的要难，遇到的困难比预料的要大，取得的成绩比预想的要好。这是县委把握大势、正确领导的结果，是县人大、县政协有效监督、全力支持的结果，是各级干部服务大局、共同努力的结果，是全县人民团结奋斗、创新拼搏的结果。在此，我代表县政府，向全县人民，向各位人大代表、政协委员和各位离退休老同志，向所有关心、支持、参与枣强发展的各界人士和朋友，致以崇高的敬意和衷心的感谢！

在肯定成绩的同时，我们也清醒地看到，我县经济社会发展中还存在着一些突出矛盾和问题。主要表现在：县域综合实力不强，经济总量不大，人均水平不高；主导产业发展水平相对较低，缺乏大企业、大项目的支撑，转型升级任务艰巨；农业基础比较薄弱，特色优势尚未形成，农民持续增收基础还不稳固；科教文卫等社会事业欠账较多，民生改善任务依然繁重；发展环境和生态环境还存在不少问题，一些部门和工作人员服务意识不强、办事效率不高，政府工作与群众期待还有差距。这些问题，我们将在今后的工作中采取有效措施，认真加以解决。

二、2013年工作安排

今年是全面贯彻落实党的十八大精神的开局之年，是为全面建成小康社会奠定坚实基础的关键一年，也是我县实现强势开局、跨越赶超的重要一年，做好今年工作意义十分重大。根据县委十届二次全委扩大会议精神，今年政府工作总的指导思想和要求是：坚持以邓小平理论、“三个代表”重要思想和科学发展观为指导，深入学习贯彻党的十八大精神和中央、省、市一系列会议精神，紧紧围绕主题主线，立足新起点，唱响主基调，实施“两步走”，谋求新跨越，努力推动枣强经济社会发展向着更高水平迈进，为全面建成小康社会奠定坚实的基础。

全县经济社会发展的主要预期目标是：生产总值增长10%左右；全部财政收入增长20%以上，其中地方一般预算收入增长20%以上；全社会固定资产投资增长23%左右，其中，固定资产投资增长24%左右；城镇居民人均可支配收入增长11%左右，农民人均纯收入增长11%左右。

实现上述目标，重点做好七个方面的工作：

（一）强势推进项目立县战略，努力在做大经济总量上开创新局面

加快项目建设速度。深入开展“双向承诺”活动，政企双方相互承诺，明确开工时间、建设期限、投资强度、推进计划，明确规划审批、清表供地、基础建设时间节点，为项目推进开辟“绿色通道”。重点抓好大营国际裘皮新城二期工程，加快推进裘都商场二期、原辅材料市场二期、品牌风尚中心、生熟皮市场、五星级酒店建设；突出抓好恒润非稳态管道、纳森空调和欧意电器北方基地项目，恒润项目年底完成主体工程，争取列入省百家重大项目，纳森空调、欧意项目年底试生产；切实抓好大营国际裘服创业园、张秀屯中小企业创业园等项目，年内取得明显形象进度。

努力提高谋划水平。开展国家产业政策和业务知识培训，提高县乡干部业务水平和项目谋划能力。围绕特色产业、基础设施、新兴产业、现代服务业、农业产业化和民生工程，谋划一批投资规模大、带动能力强的大项目、好项目。全年力争谋划亿元以上非产业类项目10个以上，其中5亿元以上项目2个以上；10亿元以上产业类项目10个以上，其中50亿元以上产业类项目1个以上。

加大招商引资力度。强化政策促激，设立专项基金，创新招商方式，大力开展特色产业定向招商、“节会”招商、上门招商、亲情招商；积极参加“5?18”廊坊经洽会、上海国际服装博览会、沈阳玻璃钢产品展览会和深圳机械制造工业展会等招商活动，努力引进一批“中”字头、“国”字号大企业、大集团，新上一批支撑性、战略性重大项目。敬众锂电与冀中能源、瑞星公司与华润集团实现合资合作。年内确保引进固定资产投资5000万元?1亿元项目3个以上，1?3亿元项目8个以上，3亿元以上项目1个以上。

着力破解瓶颈制约。坚持科学供地、集约用地，提高投资强度，叫停重复建设，严禁圈而不建、圈大建小。建立工业企业优化升级倒逼机制，综合运用环保、节能、税收等政策杠杆，努力盘活闲置资源，逐步淘汰高耗低效企业，推动优势企业提效升级。对出租出售厂房车间的企业，新上项目原则上不予供地。引导皮毛企业转变生产经营模式，科学布局功能分区，建设多层立体厂房，提高土地贡献率。创新金融产品和服务方式，完善小额贷款和信用担保公司建设，鼓励民间资本参与融资，努力帮助企业解决融资难问题。

（二）强势推进工业强县战略，努力在推动产业升级上开创新局面

制定完善产业规划。以打造产业新优势为目标，以“工业倍增”为抓手，对三大产业深度谋划，明确方向，明确定位，明确任务，明确措施，编制和完善中长期发展规划。皮毛产业，以提升加工能力、培育知名品牌、加强设计研发、开拓内外市场为重点，全力打造宜居、宜游、宜业裘皮服饰服装新名城。复合材料产业，以多基复合材料开发应用为切入点，搭建政策支持、技术研发、龙头培育平台，全力打造全国复合材料创新基地。机械制造产业，以装备高端、错位发展、区域整合为路径，建立起研发、制造、销售一体化的产业链条，全力打造现代装备制造业基地。同时，以新能源、新材料为重点，加快培育战略新兴产业，构筑新的经济增长极。皮毛、复合材料、机械制造三大产业税收力争达到2.5亿元、1亿元和8000万元。

做大做强骨干企业。筛选20家左右的骨干企业和5家左右的成长型企业，开展对标行动，强化过程帮扶，通过明确目标引领，聚集要素支持，实行增量奖励，开展订单服务，努力培育立县支柱企业。全年力争新上亿元以上技改项目40项，总投资超过40亿元；新增产值亿元企业10家、纳税千万元工业企业5家、规模以上工业企业20家；创中国名牌或驰名商标1项，省著名商标和名牌产品5项，6项产品佩挂真皮标志。恒润集团、景美化工力争全年纳税突破3000万元，上市取得实质性进展。

积极鼓励创新创业。大力推进政产学研合作，建设研发平台，开发新产品、新工艺，增强企业核心竞争力。全年力争申报市级以上科技项目15项、省级以上科技成果8项。河北省复合材料产业技术研究院、河北省输送机械研究所年内完成主体工程，河北省裘皮检测中心10月底投入使用。促进民营经济发展，积极鼓励全民创业，全年力争新增个体企业摊点1000家。

加快建设现代园区。县城工业园区，进一步扩容增效，加强基础建设，提升管理水平，增强对生产要素的承载力和吸附力。重点做好土地储备、新上项目等工作。全年力争实现产值50亿元，税收1亿元以上，新增纳税千万元企业4家，新上固定资产投资3亿元以上战略支撑项目1个。大营工业园区，进一步拉开路网框架，完成西环路北段拓宽、经贸路北延，健全完善申报条件，确保顺利升格为省级经济开发区，年内引进固定资产投资亿元以上国际国内知名企业1家。马屯工业区、秀屯工业区，在硬件设施、项目入区、管理机制等方面，制定规划，完善政策，加快建设，为工业发展搭建坚实平台。

（三）强势推进农业升级战略，努力在发展现代农业上开创新局面

全力打造农业特色。围绕农民增收目标，加快结构调整，培育蔬菜、果品和畜牧等特色优势产业，重点发展以大营、新屯、加会为主的特色养殖区，唐林、马屯为主的棚菜种植区，枣强镇、王均、恩察为主的林果种植区。认真抓好唐林省级农业示范区建设。积极推行规模化经营，重点扶持和培育3?5个3000亩以上的规模经营典型。大力实施农产品品牌创建活动，积极申报省市名牌。全年力争新增棚菜面积4000亩，达到1万亩；毛皮动物存栏达到30万只，出栏70万只。

加强农业基础建设。重点抓好卫千渠、索泸河综合治理和节水灌溉等水利工程。认真开展农业生态地质环境调查与评价，因地制宜发展优质、高产、高效的名优特农产品。千方百计稳定粮食生产，大力实施农户“万元增收”工程，全年新增“吨粮田”2万亩。切实抓好农业扶贫开发，全年新打深井20眼，安装防渗管道3万米，扶持脱贫致富示范户1000户。以乡镇政府所在地和重点村为中心，扎实推进“幸福乡村计划”，提高村民幸福指数。

提高产业化水平。创新农业经营体制，促进土地流转与规模经营相结合，每个乡镇至少建成一个土地流转面积在500亩以上的大方。积极推进农业产业化进程，重点培育一批市场前景好、增收潜力大、带动作用强的产业化龙头企业，全年市级龙头企业达到15家，力争引进省级以上农业产业化龙头企业1家。加快农民专业合作组织发展，力争全县农民专业合作社总数达到800家以上，实现行政村全覆盖；农户入社率达到20%以上，辐射带动农户率达到46%以上。筛选30家合作社重点扶持，推进公司化、规范化管理，力争10家达到省级示范社标准。

积极开展造林绿化。大力实施“一人一亩林”绿化工程，以环县城、环乡镇、环村庄绿化为重点，宜林则林，宜果则果，全年力争植树108万株，造林1.2万亩。

（四）强势推进三产兴县战略，努力在拓展现代服务业上开创新局面

做大传统服务业。以县城、大营、肖张为中心，大力发展现代物流业。谋划启动肖张物流园建设。加快餐饮业、旅店业、商贸业发展，完善服务功能，促进消费市场繁荣。推进裘皮产地市场建设，在大型商城率先推行收银结算、质量监测、价格标识、售后服务为重点的规范化管理，提升市场的层次和水平。

做强新兴服务业。全力抓好电子商务，重点打造天下裘都网、亚洲貂淘宝商城、豹王在线三大电子商务平台，拓宽营销渠道，力争网络销售额突破10亿元。积极发展旅游业，主动融入衡水湖旅游圈，精心打造“董子故里、天下裘都”地域形象品牌，大营争创国家4A级景区。积极鼓励和引导金融、评估、咨询、广告、策划、中介等服务业健康发展。

做优公共服务业。探索建立与经济发展、社会进步、城镇建设相适应的社区服务网络，加强社区、小区管理，提高物业管理水平；以建设社会主义新农村为目标，加快发展服务三农的科技网络、经纪人队伍和流通设施；积极应对人口老龄化新趋势，鼓励社会投资兴办养老机构，培育发展社会化养老服务业。

大力发展文化产业。加强董仲舒祠堂等文物保护，加快文化基础设施建设，启动枣强县文化活动中心项目，完成农村数字电视整体转换，丰富人民文化生活。启动河北空调博物馆二期工程、裘皮博物馆等文化项目建设，积极做好制裘工艺省级非物质文化遗产的申报工作。

（五）强势推进城乡统筹战略，努力在提升城镇化水平上开创新局面

完善城镇规划。按照“扩大外延、丰富内涵、完善配套、优化布局、提升功能、适度超前”要求，认真编制县域城镇规划，构建2+9（2，县城、大营；9，其他乡镇所在地）城镇发展格局。县城，按照“滨河、生态中心城市”发展定位，围绕“两轴三区”（索泸河、卫千渠，新城区、旧城区、工业区）城市框架，修订完善县城总体规划，加快编制控制性详细规划。对“一河两湖”（索泸河,吉祥湖、如意湖）等重要节点、重点区域进行高水平规划设计。大营镇，以中期建设“镇级市”、长期建成“中等城市”为目标，坚持以产兴城、产城融合，拉大框架，拓展空间，推进营新一体发展，打造优势彰显的“天下裘都”。其他乡镇，依托各自优势和风貌特征，聘请高资质单位，进行高标准规划。大营、新屯、马屯、秀屯、肖张5个乡镇，年内确保完成总体规划编制。

提升建设水平。县城，大力实施“五个一”工程，高点规划、高标建设、提升品味，把富强路打造成精品街道；加快董子公园建设，年内竣工启用；积极推进义乌小商品城项目，年内完成回迁楼建设，综合商城主体竣工；规划启动董子文化长廊、第二污水处理厂等公共设施建设，加快农贸市场规划建设和人民街改造，完成汽车站建设搬迁；扎实推进集中供热和天然气进城，年底集中供热面积达到150万平方米，县城燃气普及率达到50%以上。大营镇，重点实施裘皮新城路网工程，完成天然气进城，启动第三水厂，力争成为全市综合配套改革试点镇、示范镇。张秀屯乡、新屯乡积极推进撤乡建镇工作，力争年内完成。今年，全县城镇化率力争达到50%。

延伸城市管理。紧紧围绕建管水平双提升目标，加快推进城区管理“网格化”、城乡环卫“一体化”、市容管理“精细化”、广告管理“标准化”、城市管理“数字化”建设；继续抓好城市综合整治和违章建筑专项治理，治理范围向小区延伸、向城中村延伸、向乡镇延伸，年内完成肖张、王均、新屯等沿路集贸市场治理整顿。以“四清四化五改”为主要内容，全面治理农村脏、乱、差问题，逐步建立农村环境综合整治长效机制。重点抓好大营镇?新屯乡、枣强镇两个片区的整治工作。全县11个乡镇全面建立城镇建设管理体系，做到有机构、有人员、有保障，力争垃圾无害化处理率达到100%。

争创省级园林城。重点做好森林公园扩建工程；加快“一河两湖”建设进度，着力做好湖区绿化；加强单位庭院和居住小区绿化，千方百计增加绿量，主干道绿地率达到20%，次干道绿地率达到15%，确保省级园林县城创建成功。

（六）强势推进民生优先战略，努力在促进和谐稳定上开创新局面

全力保障和改善民生。实施更加积极的就业政策，确保新增就业5000人以上；巩固扩大社会保障，努力实现城乡居民社会养老保险全覆盖；健全社会救助体系，做好救灾救济、优抚安置、城乡低保、农村五保、流浪救助等工作；加强住房保障，完成670套保障性住房和2900套农村危房改造任务。加快实施肃临线南段升级改造工程，10月底竣工通车；完成王均?加会段和50公里农村公路建设；改建和维修仝庄桥、王杨兴桥等6座农村桥梁；深入开展基层建设年项目帮扶，认真做好55个村街道硬化等10件实事。启动建设肖张、张秀屯两个水厂，扩大联村供水面积，健全完善水厂运行机制；抓好南水北调配套工程，切实做好沿途乡镇引蓄水工作。

协调发展社会各项事业。鼓励和引导民间资本参与发展医疗事业、教育和社会培训、社会福利事业。加大教育投入，大力改善办学条件，缩小城乡、校际差距，推进义务教育均衡发展。积极解决县城中小学班容量过大的问题，完成第三中学和特教学校建设，成立第四小学，确保新学年投入使用。加快新县医院建设进度，年内完成主体工程；完成枣强镇、肖张镇、王常乡卫生院扩建工程和100所农村卫生室新建任务，完善县中医院建设。继续抓好农村合作医疗和城镇居民基本医疗，深入推进医药卫生体制改革，实现县级公立医院药品零差率销售。加强人口和计划生育工作，严格落实目标管理责任制，加快后进转化，稳定低生育水平。实施全民健身工程，积极开展各类体育活动。大力发展妇女儿童、红十字等事业，全力做好关心下一代、老龄、残疾人、群团、双拥、人民武装等工作。

积极推动社会管理创新。严格落实信访工作责任制，妥善解决群众合理诉求。深入开展安全生产专项整治活动，坚决查处各类非法违规生产经营行为。积极稳妥推进企业改制，切实维护职工利益。加强公共卫生、食品安全、社会安全事件的预防和应急处理，提高应对突发事件能力。顺应群众对公共安全的新期待，加快构建全县无缝隙信息网络体系，严厉打击各类违法犯罪活动，全力建设平安枣强，不断增强群众的安全感和幸福感。

（七）强势推进环境保障战略，努力在优化发展环境上开创新局面

构建高效的政务环境。加强政务服务中心建设，逐步推行网上审批，提高政府行政效能。全面清理行政审批事项，及时公布清理精简结果。推行行政审批“十公开”制度，即公开“审批主体、审批内容、审批依据、审批条件、审批程序、审批时限、收费标准、工作纪律、投诉渠道、责任追究”等十项内容，缩短办结时限，提高办事效率。

构建宽松的政策环境。全面落实对企业扶优扶强的意见、招商引资和项目建设奖励办法等政策文件，为企业发展提供有力的政策保障。积极探索实行涉企收费“一费制”，推行“一口受理、一章审定、一费缴清”扎口式管理模式。严格规范行政处罚自由裁量权和行政性收费标准，做到低限收费、下限收取，努力做到政策最优惠、环境最宽松。

构建优质的服务环境。探索推行涉政事务代办制，对投资项目代理代办、联审联批。成立公共资源交易中心，打造规范有序、高效透明的公共交易平台。实行“企业安静生产经营日”制度，确保企业高效生产经营。定期召开企业恳谈会，倾听企业意见，了解企业困难，协调解决企业实际问题。

构建良好的生态环境。坚定不移走绿色发展、循环发展、低碳发展之路。扎实抓好节能减排，强化源头控制，认真执行环评、能评和“三同时”制度，严格控制高耗能、高污染项目。加强PM2.5监测治理，力争空气质量达到国家新标准。开展治污专项行动，下大力解决好重点行业、重点区域的污染现象，切实改善群众生活环境。

三、全面加强政府自身建设

面对新的形势和任务，必须把思想和认识统一到党的十八大精神上来，统一到省市的重大决策部署上来，统一到县委“凝神聚力谋发展、真抓实干惠民生”的总要求上来，认真执行改进工作作风、密切联系群众的八项规定，切实做到为民、务实、清廉，全力建设人民满意的政府。

全力建设以人为本、爱民亲商的服务型政府。坚持把群众需求作为引领政府工作的根本导向，零距离倾听群众呼声，面对面感受群众需要，心连心重视群众疾苦。坚持把客商满意作为评判政府工作的重要标尺，对投资者的承诺，定一条抓一条，条条算数；说一项干一项，项项兑现。

全力建设锐意改革、创新有为的务实型政府。抓住衡水纳入首都经济圈发展规划和省级综合配套改革实验区的机遇，以改革强基础，以创新增活力，靠实干创造实绩、凭实绩赢得民心。切实增强政府执行力和落实力，把任务项目化、项目目标化、目标节点化、节点责任化，加快重点工作、重要项目的推进落实。

全力建设依法行政、规范透明的阳光型政府。自觉接受人大法律监督、工作监督、政协民主监督和社会公共监督，认真做好代表委员议案提案办理工作。全面推行行政执法责任制，加强执法队伍建设，完善行政执法程序，把依法行政落实到政府工作的各个方面。完善重大决策的咨询、听证、公示和法律顾问机制，努力提高政府决策的科学化、民主化、法治化水平。

全力建设克己奉公、风清气正的廉洁型政府。坚定理想信念、严守政治纪律、把住廉政底线、不谋私利特权，切实做到干部清正、政府清廉、政治清明。落实党风廉政建设责任制，完善惩治和预防腐败体系建设，更加注重从源头上预防和治理腐败。坚持和发扬艰苦奋斗精神，严格执行公务接待制度，严格落实各项节约措施，把更多的资金用到事业发展和改善民生上。以勤政为民、廉洁清正的实际行动，树立党和政府的良好形象。

各位代表，枣强正处在新的历史起点，面对新目标新任务，我们深感责任重大、使命光荣。让我们在党的十八大精神指引下，在中共枣强县委的正确领导下，凝神聚力，真抓实干，强势开局，跨越赶超，为全面建成小康社会而努力奋斗！
